# Adenomyoepithelioma: A Case Report of a Rare Breast Lump

**DOI:** 10.7759/cureus.62931

**Published:** 2024-06-22

**Authors:** Sudhir Jayakar, Siddharth Tiwari, Kondapalli Sri Sai Teja Sampath, Guneet Singh, Vinay Badangi

**Affiliations:** 1 General Surgery, Dr. D. Y. Patil Medical College, Hospital and Research Centre, Dr. D. Y. Patil Vidyapeeth (Deemed to be University), Pune, IND

**Keywords:** benign, cytology, fibroadenoma, young age, breast adenomyoepithelioma

## Abstract

Adenomyoepithelioma (AME) of the breast is a rare tumor characterized by biphasic differentiation into luminal and myoepithelial cells, with various histological patterns observed. This case report details a 35-year-old female with a progressively enlarging breast lump diagnosed initially as a fibroadenoma through ultrasonography (USG) and fine-needle aspiration cytology (FNAC). The patient underwent successful excision of the lump under general anesthesia, with histopathological examination confirming a benign tumor comprising epithelial and myoepithelial cells. This case underscores the importance of comprehensive clinical assessment and accurate diagnostic techniques in managing breast lumps, emphasizing the need for timely intervention for favorable outcomes.

## Introduction

Adenomyoepithelioma (AME) of the breast, a rare tumor first described in 1970, exhibits variable histopathological behavior [1,2]. While the majority are benign, they often have a propensity for recurrence. Malignancy has been reported in up to 40 cases, indicating the potential for adverse outcomes. Despite the rarity of these tumors, they pose diagnostic challenges as imaging studies are inconclusive and typically do not provide definitive diagnoses [3].

AME displays a wide variety of histological patterns, both within and among individual tumors, and shows a characteristic biphasic differentiation into luminal cells and myoepithelial cells [4,5]. This heterogeneity makes them difficult to diagnose, even with core needle biopsies. In the majority of cases, these tumors are centrally located within the breast tissue, often in close proximity to or within the areola [6]. This anatomical distribution can be attributed to the histological arrangement of myoepithelial cells, which are situated between the ducts and the basal layers of the acinus within the breast tissue. The areolar region, where the major ducts of the breast converge, contains a rich abundance of myoepithelial cells, making it a common site for the development of AME [7].

While most tumors follow a benign clinical course, the potential for local recurrences and malignant transformations has also been reported [5]. In cases where malignancy is suspected, it is imperative to recognize significant markers including cytologic atypia and brisk mitotic rates. To identify these features and guide appropriate treatment strategies the sampling of the tumor should be adequate. Thorough excision, including sufficient margins, is recommended to minimize the risk of local recurrence and potential metastasis [3]. Close monitoring and follow-up postoperatively are essential to promptly detect any signs of recurrence or metastasis and ensure timely intervention for optimal patient outcomes.

## Case presentation

A 35-year-old female presented to our outpatient department with a chief complaint of a lump in her left breast that she first noticed a year ago. Over the past four months, the lump has progressively increased in size from approximately 1x1 cm to its current size of 5x5 cm. The onset of the swelling was insidious, and the patient did not experience associated pain or any discharge from the nipple. The patient has two children who were breastfed and reported regular menstrual cycles. There was no history of trauma or consumption of oral contraceptive pills. Upon general examination, her vitals were stable. Local examination revealed a normal right breast, while the left breast exhibited a 5x5 cm lump in the lower outer quadrant. The lump was freely mobile, globular in shape, with a bosselated surface, and hard in consistency. No signs of peau d'orange, engorged vessels, or nipple retraction were observed. Additionally, the nipple-areola complex appeared normal, and there was no evidence of axillary or supraclavicular lymphadenopathy.

Systemic examination revealed no abnormalities detected (NAD). The patient's blood group was O-positive. Laboratory investigations showed a hemoglobin level of 9.30 g/dL, total leukocyte count (TLC) of 6370 cells/μL, and platelet count of 321,000 cells/μL. The prothrombin time (PT) was 13.50 seconds, and the international normalized ratio (INR) was 1.14 within normal limits. Total protein was measured at 6.90 g/dL, with albumin levels at 4.10 g/dL and globulin levels at 2.80 g/dL, resulting in an albumin-to-globulin ratio (A:G) of 1.46. Renal function parameters indicated urea levels of 20 mg/dL and creatinine levels of 0.63 mg/dL. Electrolyte levels were within normal ranges, with sodium at 137 mEq/L, potassium at 4.02 mEq/L, and chloride at 104 mEq/L. The reference ranges are presented in Table 1.

**Table 1:** Laboratory investigations Hb: Haemoglobin; TLC: Total leucocyte count; PT/INR: Prothrombin time/international normalized ratio, A:G: Albumin-to-globulin ratio

Parameter	Observed Value	Reference Value
Hb	9.30	13.2-16.6 g/dL
TLC	6370	4000-10000 μL
Platelet	321000	150000-400000/μL
PT/INR	13.50/1.14	10.24-12.71s/0.85-1.15
Protein (Total)	6.90	6.4-8.3 g/dL
Albumin	4.10	3.5-5.2 g/dL
Globulin	2.80	2.3-3.5 g/dL
A:G	1.46	-
Urea	20	17-49 mg/dL
Creatinine	0.63	0.6-1.35 mg/dL
Sodium	137	136-145 mmol/L
Potassium	4.02	3.50-5.10 mmol/L
Chloride	104	98-107 mmol/L

The patient underwent ultrasonography (USG) of both breasts and axilla, revealing a well-defined, well-circumscribed, heterogeneously hyperechoic lesion measuring 28x37 mm with internal vascularity, suggestive of a fibroadenoma (Breast Imaging-Reporting and Data System (BI-RADS) 2 lesion). Further confirmation was obtained through fine-needle aspiration cytology (FNAC) of the left breast, which was consistent with fibroadenoma.

**Figure 1:**
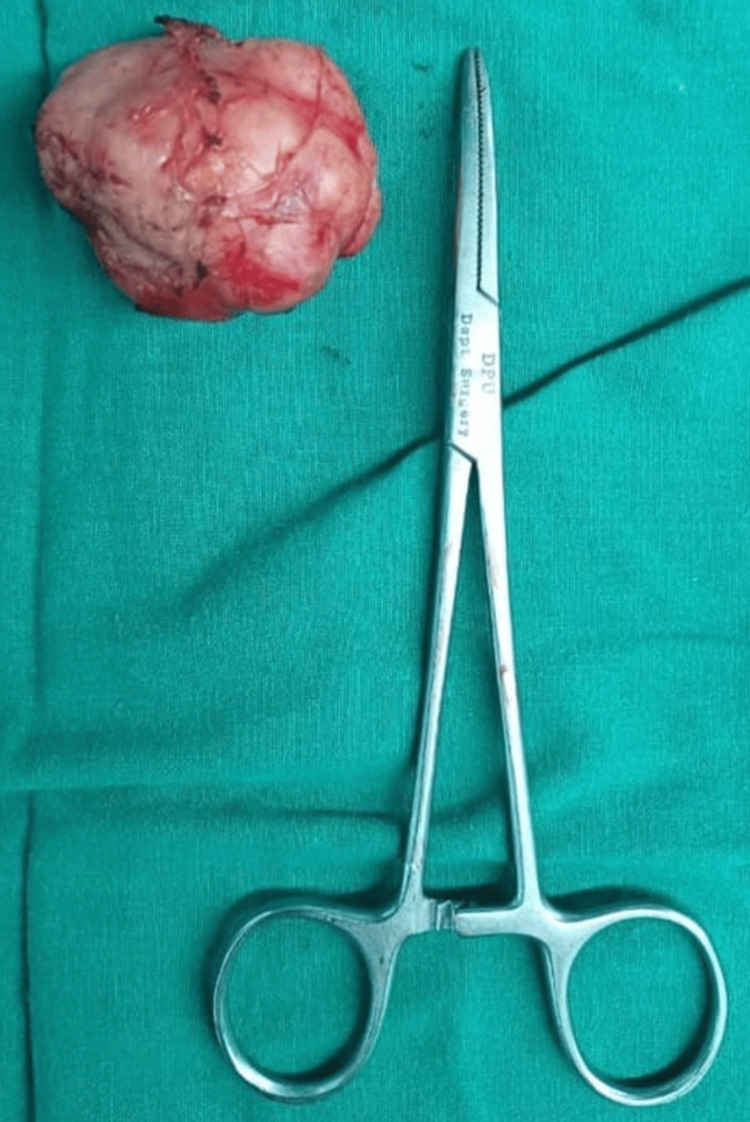
Excised specimen of the left breast mass

**Figure 2:**
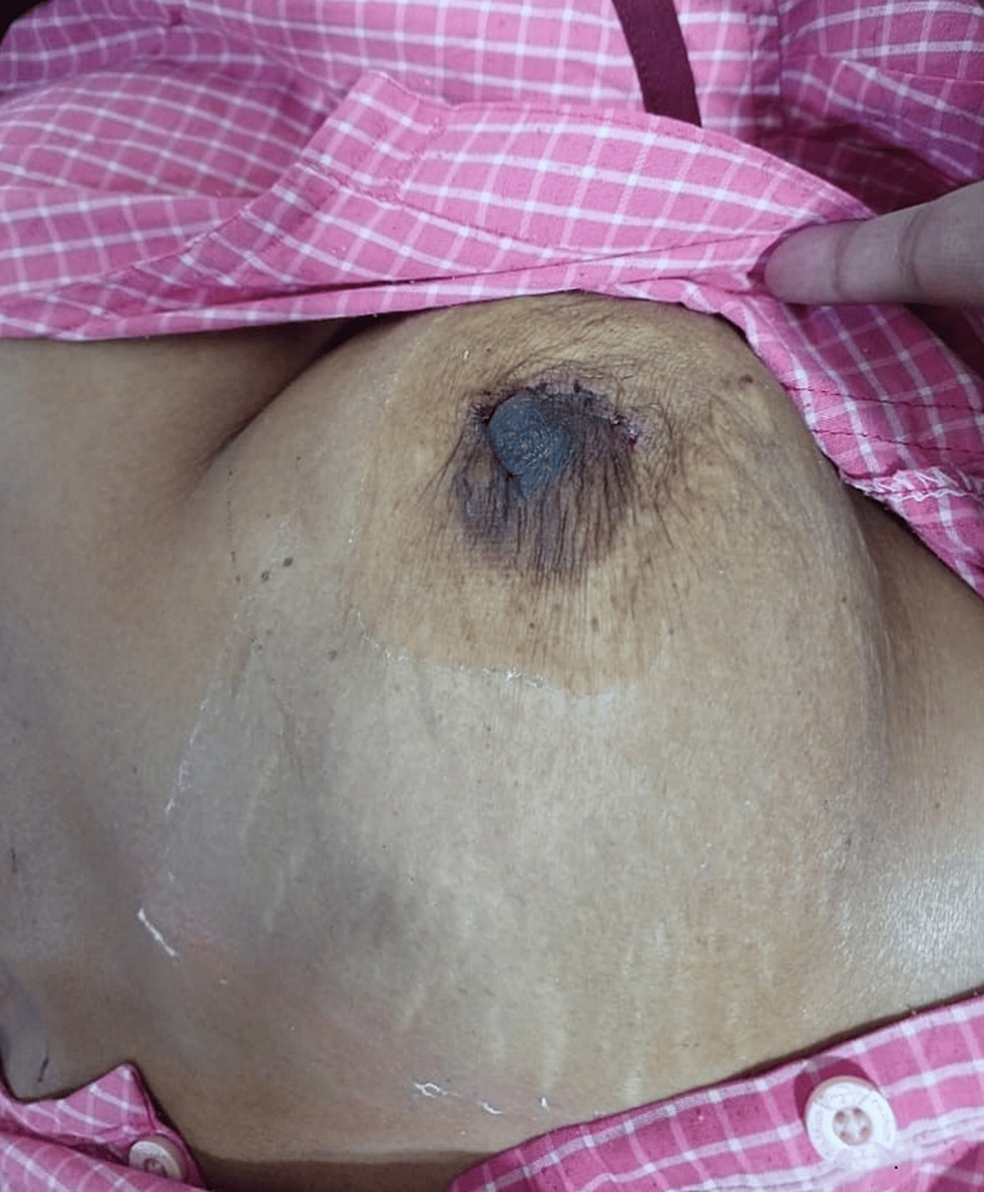
Postoperative day three

Subsequently, the patient underwent excision of the lump under general anesthesia. A 3 cm circumareolar incision was made at the 2-4 o’clock position. The tissue was dissected, and the capsule of the mass was identified and circumferentially freed from adhesions. The mass was excised as a whole and sent for histopathological examination (Figure 1). Hemostasis was achieved, and closure was performed using monocryl 3-0 sutures. The patient was discharged on postoperative day five after careful monitoring during the entire hospital course (Figure 2).

**Figure 3:**
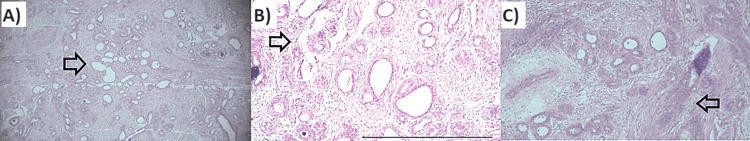
Histopathological images (A) Numerous myoepithelial cells; (B) Glandular cells with bordering epithelial cells; (C) Muscular and glandular cells in the specimen

Histopathological examination (Figure 3) of the excised mass revealed multiple sections of a well-circumscribed benign tumor composed of a biphasic proliferation of epithelial and myoepithelial cells. The epithelial cells were arranged in glandular patterns with some areas showing cystic dilation while the surrounding myoepithelial cells exhibited polygonal shapes with clear cytoplasm. Additionally, the stroma exhibited fibrosis. No evidence of atypia or malignancy was observed in the histopathology report, confirming the diagnosis of AME instead of the initial finding of fibroadenoma.

## Discussion

AME is an uncommon breast tumor that predominantly affects females, with very rare reported cases in males to date [8,9]. Although there is no fixed age distribution for its occurrence, patients are typically diagnosed in middle age or older, with cases reported in individuals ranging from 16 to 86 years old [8]. While the majority of known cases present with symptomatic breast lumps, a minority may remain asymptomatic, with the tumor being incidentally detected during routine imaging or self-examination. In some instances, patients initially diagnosed with benign breast masses may later seek secondary opinions due to persistent symptoms or suspicion of misdiagnosis, as observed in our patient, possibly attributed to sampling errors or misinterpretation of imaging findings [10].

In the majority of documented cases, AME is unilateral, affecting only one breast. However, a notable exception was reported by Bajpai et al. in 2013, detailing a 16-year-old girl diagnosed with bilateral AME [11]. While AME typically manifests as solitary masses within the breast tissue, there have been instances where patients presented with multiple adjacent masses. In one such case, both lesions were identified through mammography, highlighting the importance of comprehensive imaging studies in detecting and characterizing these tumors [12,13].

Malignant transformation of AME is rare, with only 40 documented cases reported to date. While the metastatic potential of these tumors is still being studied, available literature suggests that metastases primarily occur via hematogenous dissemination rather than through the lymphatic system. Notably, metastasis tends to occur in tumors larger than 2 cm in size [3].

Histologically, AME exhibits significant heterogeneity, which can pose challenges in accurate diagnosis [2]. In some cases, casual tissue collection methods, such as FNAC, may yield misleading results [9]. For instance, initial FNAC findings in our case incorrectly indicated a diagnosis of fibroadenoma. Due to the hypercellular nature of these tumors, cytological methods alone may be insufficient and unreliable for definitive diagnosis. A more reliable approach to diagnosing AME involves obtaining a core biopsy, which allows for a more detailed assessment of histopathological features [14]. Unlike FNAC, core biopsy provides a larger tissue sample, enabling pathologists to better evaluate the cellular composition and landscape of the lesion. This method facilitates the identification of the cellular elements characteristic of AME, ultimately leading to a more accurate diagnosis.

## Conclusions

AME of the breast is a rare benign condition with the possibility of becoming malignant. Accurate diagnosis relies heavily on clinical suspicion and close correlation with pathological findings. Typically, these tumors exceed 2 cm in size and do not spread to the axillary lymph nodes, as their dissemination occurs through the bloodstream. Diagnostic procedures like USG and FNAC often lack reliability, making core biopsy the preferred method for obtaining a definitive diagnosis. Definitive management encompasses excision of the tumor, and regular patient follow-up is essential due to the potential for tumor recurrence and varying degrees of malignant potential.
